# Construction of subunit vaccine by fusing *Salmonella* flagellin with neutralizing epitopes of porcine epidemic diarrhea virus

**DOI:** 10.3389/fvets.2026.1827170

**Published:** 2026-07-15

**Authors:** Xudong Gao, Yuanfang Rao, Aiqing Jia, Guiping Wang, Jinming Cui

**Affiliations:** 1 Animal Husbandry and Aquatic Research Center, Guangdong Haida Group Co., Ltd., Guangzhou, Guangdong, China; 2 Guangdong Provincial Key Laboratory of Pig Breeding and Disease Control Technology, Guangzhou, Guangdong, China; 3 Guangdong Hairui Biotechnology Co., Ltd., Guangzhou, Guangdong, China

**Keywords:** flagellin, porcine epidemic diarrhea, porcine epidemic diarrhea virus, subunit vaccine, swine

## Abstract

**Introduction:**

To address practical application and production demands, this study aims to develop a Porcine epidemic diarrhea (PED) subunit vaccine with high expression efficiency and immunogenicity, using molecular biology and structural biology approaches.

**Methods:**

Based on the established immunostimulatory properties of flagellin, a recombinant fusion protein was constructed by linking the *Salmonella choleraesuis* flagellin mutant FliC_L3A_ to the core neutralizing epitope (COE) of the spike protein of porcine epidemic diarrhea virus (PEDV). The target protein was expressed using a CHO-S cell expression system and subsequently formulated into a subunit vaccine with adjuvant 201. Immunogenicity was systematically evaluated in a mouse model by measuring serum levels of PEDV-specific IgG, IgA, and IgM antibodies, as well as the cytokines IFN-*γ* and IL-4.

**Results:**

Structural prediction of the FliC_L3A_-COE fusion protein by AlphaFold2 indicated that the protein can spontaneously assemble into a bouquet-like multimeric structure. Following condition optimization, a CHO-S cell expression system with a transfection efficiency of approximately 80% was successfully established. Post-immunization ELISA results demonstrated that the level of PEDV-specific IgG increased progressively with the number of immunizations and reached a peak after the second booster immunization; IgA levels peaked after the first booster immunization but declined following the second booster; IgM levels reached their maximum after the primary immunization and decreased significantly with subsequent boosters. The expression trends of IFN-*γ* and IL-4 were consistent, both continuously increasing with each booster immunization. Compared with the commercial vaccine, the FliC_L3A_-COE subunit vaccine induced lower levels of IFN-*γ*; the levels of IgG, IgA, IgM, and IL-4 showed no significant difference from those induced by the commercial vaccine.

**Conclusion:**

The FliC_L3A_-COE fusion protein induces PEDV-specific antibodies and elicits detectable immune responses in mice. Its immunogenicity profile in terms of antibody induction is generally similar to that of a commercial vaccine, although IFN-*γ* responses were lower. These findings indicate that the fusion protein has certain immunostimulatory properties, but further optimization may be needed to improve its immune potency.

## 1 Introduction

Since its initial discovery in 1971 and subsequent introduction into China in 1984, Porcine Epidemic Diarrhea (PED) has incurred substantial economic losses and remains a major threat to the global swine industry (1). PED is a highly contagious intestinal disease caused by PED virus (PEDV) infection, clinically characterized by severe watery diarrhea, vomiting, and dehydration. While PEDV can infect pigs of all ages, neonatal piglets are particularly susceptible due to their underdeveloped immune systems. Notably, the mortality rate can reach 100% in infected piglets within 0–7 days of age, making disease control at this stage critically important (2).

PEDV belongs to the genus *Alphacoronavirus* within the family *Coronaviridae*. The enveloped virion contains four major structural proteins: the spike (S) protein, membrane (M) protein, envelope (E) protein, and nucleocapsid (N) protein. The viral genome comprises open reading frames ORF1a and ORF1b, which encode replicase polyproteins essential for viral replication. Additionally, ORF3 encodes an accessory protein that functions as an ion channel and may participate in immune modulation. Collectively, these viral proteins play crucial roles in host cell invasion and the pathogenesis of PEDV (3–7). The S protein plays a critical role in virus–host cell interactions, primarily mediating membrane fusion and the specific recognition events associated with neutralizing antibodies. The S protein is composed of two subunits, S1 and S2. The S1 subunit exhibits high antigenicity and an elevated mutation rate, functioning as the principal receptor-binding domain and the primary site of antigenic recognition. The C-terminal domain (CTD) of the S1 subunit, which serves as the major target region for neutralizing antibodies, has been defined as the core neutralizing epitope (COE) (8–10).

In practice, controlling PED in piglets primarily relies on passive immunity conferred by maternal antibodies. Sows transfer specific antibodies, predominantly secretory immunoglobulin A (sIgA), to piglets via colostrum and milk. Upon ingestion, these antibodies localize to the intestinal mucosa, providing local immune protection. The production of sIgA is mainly stimulated by the sow’s mucosal immune response. Due to its high transmissibility and pathogenicity, PEDV poses significant control challenges. Vaccination remains the primary prevention strategy, with inactivated and live-attenuated vaccines being commonly used (11, 12). However, vaccines administered via intramuscular injection have a limited capacity to induce robust mucosal immune responses and often fail to generate sufficient levels of sIgA. Consequently, the transfer of protective antibodies to piglets through colostrum is suboptimal. Furthermore, the high mutation rate of the PEDV genome leads to antigenic variations among different strains, resulting in limited cross-protective efficacy of existing vaccines against circulating field variants and further complicating control efforts (13).

To enhance immunoprotective efficacy, various novel vaccine strategies have been proposed. Studies have shown that a subunit vaccine, developed using a fusion protein of the PEDV S protein S1 domain and a *Salmonella* flagellin mutant (FliC_L3A_), can efficiently induce mucosal immune responses and promote the production of sufficient sIgA when administered to sows via a mucosal route (14, 15). Furthermore, fusing the S protein with a trimerization domain promotes the formation of a trimeric conformation *in vitro*, which more closely mimics its native structure on the viral surface. Compared to S protein monomers, this trimeric conformation has been demonstrated to more effectively elicit neutralizing antibodies and immune responses (16, 17). Although current subunit vaccines are primarily in the laboratory research stage and have not yet entered clinical application, they offer significant advantages such as ease of engineering, functional versatility, and high safety, positioning them as a promising direction for future vaccine development.

This study describes the development of a subunit vaccine against PEDV. The vaccine was designed by fusing the core neutralizing epitope (COE) from the viral S1 protein with *Salmonella choleraesuis* flagellin, with the aim of inducing enhanced mucosal immunity.

## 2 Materials and methods

### 2.1 Primer synthesis and sequencing

Gene synthesis, primer synthesis, and plasmid sequencing services were provided by Sangon Biotech (Shanghai) Co., Ltd. The complete gene sequence of the S protein of a G2b subclade PEDV strain (CH/JSLYG/02/2021) was obtained from the published NCBI database (GenBank accession No. MZ161072.1), and its COE region was defined as amino acid residues 499–638. The sequence of *Salmonella choleraesuis* flagellin was retrieved from the published NCBI database (GenBank accession No. WP_086810749.1). The pCAG eukaryotic expression vector was kindly provided by Professor Guanghui Yang (College of Biological Sciences, China Agricultural University). Primer design was performed using SnapGene software (see Table 1).

**Table 1:** Primer for S complex protein gene amplification of PEDV strain.

Primer name	Nucleotide sequence (5′ → 3′)	T_m_ (°C)	Length of amlplification
pCAG-F	TAAAAGCTTGCTAGCAGATCTTTTTCCCTC	61	7,746
pCAG-R	GGTGGCGGTACCTCCGGATCGATA	66	
His(pCAG)-F	GGTACCGCCACCATGCACCACCACCACCATCAC	72	786
TEV(FliC)-R	GGCTGAACCTCTAGAGCCCTGAAAATACAGGTT	66	
FliC(TEV)-F	TTTCAGGGCTCTAGAGGTTCAGCCCAGGTCATA	69	2010
COE (pCAG)-R	GCTAGCAAGCTTTTACACCCCCTCCAGTGGTTT	68	

### 2.2 Protein structure design and prediction

This study utilized the PEDV G2b subtype strain CH/JSLYG/02/2021, isolated in Lianyungang City, Jiangsu Province, China, in 2021. The COE region (499–638 aa) within the viral S protein was selected as the primary antigenic site. A fusion protein was engineered by linking the COE sequence to the C-terminus of a mutated *Salmonella choleraesuis* flagellin FliC_L3A_ via a GS linker.

The 3D structure of the target protein was predicted using the online tool AlphaFold2 (AlphaFold2.ipynb - Colab) and subsequently visualized and analyzed with the PyMOL software.

### 2.3 Cloning of the target gene

To achieve high-yield expression of the target protein in the CHO expression system, the coding gene sequence was first optimized according to CHO codon usage bias and subsequently synthesized entirely by Sangon Biotech (Shanghai). Following the acquisition of the optimized gene fragment, a series of molecular constructs were engineered. Specifically, a Tobacco Etch Virus (TEV) protease cleavage site (amino acid sequence: ENLYFQG) was incorporated at the N-terminus of the target protein to facilitate future tag removal. Upstream of this cleavage site, an Enhanced Green Fluorescent Protein (EGFP) tag was fused to enable real-time monitoring of transfection efficiency. Furthermore, a 6 × His-tag was added to the N-terminus of EGFP for purification via nickel-affinity chromatography. Finally, this multi-component fusion protein construct was cloned into the eukaryotic expression vector pCAG, resulting in a recombinant plasmid suitable for transient expression in mammalian cells.

### 2.4 CHO-S cell culture conditions

CHO-S cells were cultured in CD01-CHO serum-free medium (Jiangsu Baijiete Biotechnology Co., Ltd.) under conditions of 130 rpm, 37 °C, and 5% CO₂. Cell density and viability were monitored daily using a cell density analyzer. When the cell density reached 2 × 10^6^ to 3 × 10^6^ cells/mL and viability exceeded 95%, the cells were subcultured at a split ratio of 1:2.

### 2.5 Cellular instantaneous transfection

Transient transfection of CHO-S suspension cells was performed using a liposome-mediated method. Transfection was initiated when the cell density reached 2–4 × 10^6^ cells/mL. A transfection complex was prepared at a ratio of 10 μg of plasmid DNA and 20 μL of liposomal transfection reagent per 1 × 10^7^ cells. The detailed procedure was as follows: first, an appropriate amount of the target gene plasmid was diluted in 1 mL of antibiotic-free medium. Simultaneously, a corresponding volume of the liposomal transfection reagent was mixed with an equal volume of antibiotic-free medium, also adjusted to a final volume of 1 mL, and incubated at room temperature for 5 min. Subsequently, the plasmid mixture and the liposome mixture were combined, gently mixed, and incubated at room temperature for 20 min to allow for DNA-liposome complex formation. Finally, the entire 2 mL of complex solution was added to the CHO-S cell suspension. The cells were then cultured under standard conditions (37 °C, 5% CO₂) with orbital shaking at 130 rpm. The cells were harvested 60 h post-transfection for subsequent protein purification.

### 2.6 Protein purification

The target protein was purified from CHO-S cell pellets by Ni-NTA affinity chromatography. Cell pellets were resuspended in lysis buffer (150 mM NaCl, 25 mM Tris–HCl, pH 8.0) and subjected to ultrasonication on ice using an ultrasonic cell disruptor (JY92-IIN, Φ6 mm probe; Ningbo Scientz Biotechnology Co., Ltd.) with the following parameters: power output of 300 W, pulse cycle of 10 s on / 10 s off, and a total sonication time of approximately 3 min. The sample temperature was maintained below 25 °C throughout the process. After centrifugation (12,000 × *g*, 30 min, 4 °C), the supernatant was incubated with Ni-NTA resin. The column was washed with buffer containing 30 mM imidazole, and the protein was eluted with buffer containing 300 mM imidazole.

To remove the fusion tag, TEV protease was added at a protease-to-substrate mass ratio of 1:10 (w/w), and the mixture was incubated at 4 °C for 16–18 h. The cleavage reaction mixture was subsequently passed through a Ni-NTA affinity column a second time to separate the tag-free target protein from the TEV protease and any uncleaved fusion protein, thereby yielding highly purified, tag-free protein.

The tag-free protein was further purified by size-exclusion chromatography (SEC) on a Superose 6 10/300 column equilibrated with 150 mM NaCl and 25 mM Tris–HCl (pH 8.0). Peak fractions corresponding to the target protein were identified by SDS-PAGE, pooled, and concentrated to obtain the final purified protein.

### 2.7 Vaccine preparation

Following the acquisition of the target protein at a concentration of approximately 200 μg/mL, the vaccine formulation was prepared. In this study, the 201 adjuvant was selected as an immunopotentiator and emulsified with the target protein at a 1:1 volume ratio. The specific procedure was as follows: within a biosafety cabinet, the protein solution, equilibrated to room temperature, was transferred to a sterile beaker. An equal volume of the 201 adjuvant was then added and mixed gently. A sterilized magnetic stir bar was placed into the beaker, which was subsequently positioned on a magnetic stirrer. Emulsification was carried out at 31 °C with continuous stirring at 300 rpm for 10 min to form a homogeneous emulsion. After stirring, the final formulation was aseptically aliquoted and stored at 4 °C for subsequent use. Strict aseptic techniques were maintained throughout the entire procedure and during subsequent handling to ensure the stability and reliability of the vaccine preparation.

### 2.8 Mouse immune experiment

Male BALB/c mice aged 6–8 weeks were randomly assigned into three groups (n = 3 per group) using a random number table: the experimental group, the PBS negative control group, and the positive control group receiving the commercial inactivated vaccine Kexunning (KXN). Mice were immunized via multipoint subcutaneous injection in the neck and thigh regions for a total of three immunizations administered at 14-day intervals, with a single injection volume not exceeding 200 μL per mouse. Tail vein blood samples were collected 1 week after each immunization, and terminal blood samples were obtained by retro-orbital bleeding 2 weeks after the third immunization. The entire experiment was independently repeated twice, and statistical analysis was performed to evaluate potential batch effects between the two experimental replicates to exclude any confounding influence of inter-batch variability on the results.

Serum was separated from the collected blood samples by centrifugation. The levels of PEDV-specific IgG, IgA, and IgM antibodies in the serum were measured using commercial ELISA kits. Furthermore, the concentrations of Th1/Th2 cytokines, including interferon-gamma (IFN-*γ*) and interleukin-4 (IL-4), in the mouse serum were determined using a cytokine detection kit to systematically evaluate the vaccine-induced humoral and cellular immune responses.

A portion of the mouse immunization experiments was outsourced to and performed by the Beijing Qingxi Technology Research Institute.

## 3 Results

### 3.1 Subunit vaccine design and purification

**Figure 1:**
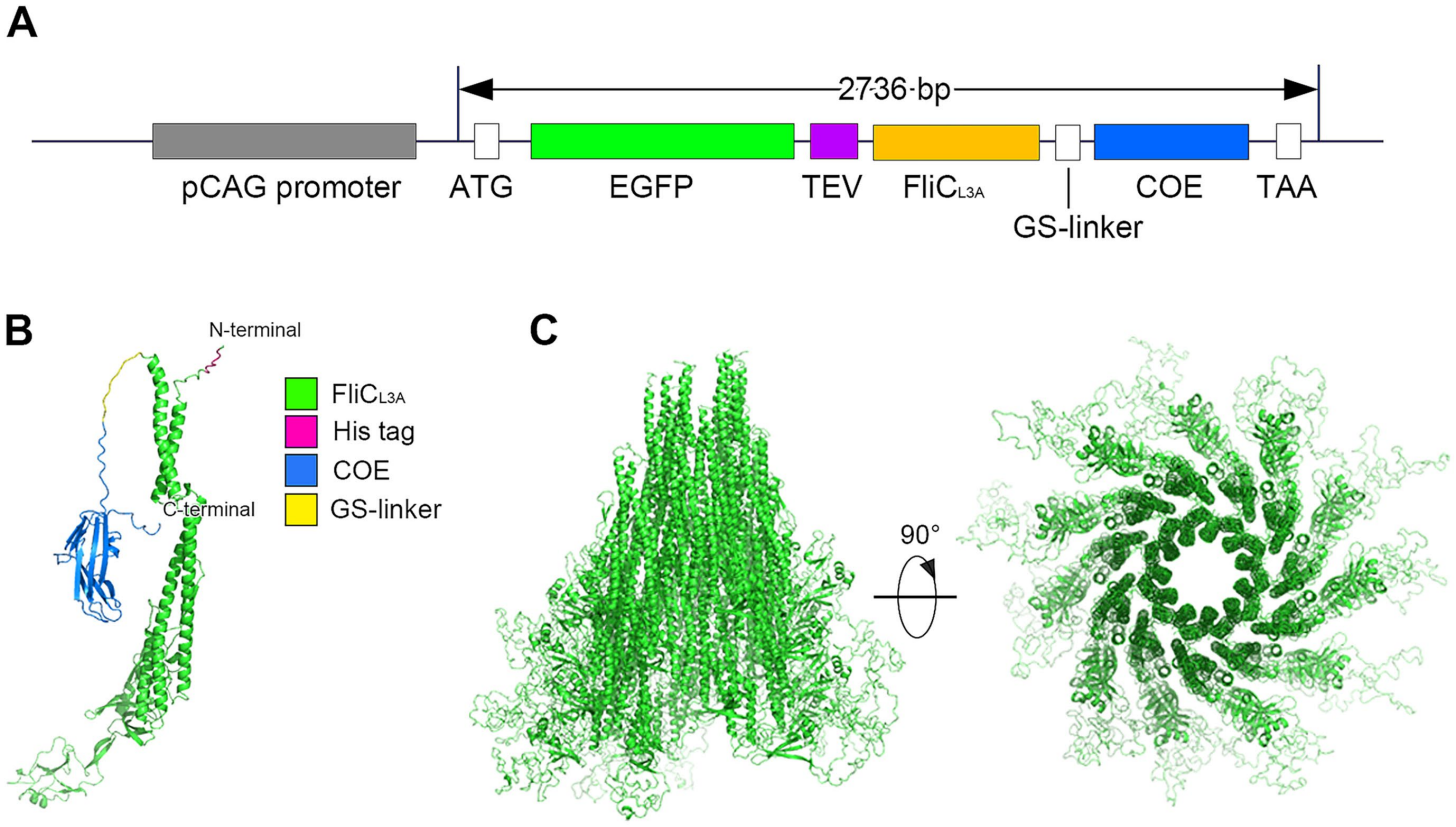
Predicted structure of the fusion protein. **(A)** Gene expression cassette diagram of fusion protein. **(B)** Side view of the fusion protein monomer structure. **(C)** Structure of the flagellin multimer. The left panel shows a side view, and the right panel shows a top view. All structures were predicted using AlphaFold 2.

Currently, the predominant PEDV subtypes circulating in China are the G2a and G2b sublineages. Based on epidemiological surveillance, this study selected the CH/JSLYG/02/2021 strain identified in Jiangsu Province in 2021 (Accession No. MZ161072.1) as the research subject. The COE region within the S protein of this strain was engineered for protein development. Meanwhile, the flagellin protein FliC from *Salmonella choleraesuis* (Accession No. WP_086810749.1) was mutated at residues L503A, L505A, and L506A. The COE region was fused to the C-terminus of the resulting mutant, FliC_L3A_, to generate a fusion protein with enhanced immunogenicity. The schematic diagram of the protein structure is shown in Figure 1A, and the prediction of the composite protein structure is presented in Figure 1B. Previous studies have shown that FliC can self-assemble into multimers with a bouquet-like structure. We hypothesize that the fusion of the COE region to the C-terminus of flagellin facilitates the formation of polymerized multimeric structures, which may enhance protein immunogenicity and effectively trigger pathogen-associated molecular pattern (PAMP) responses, thereby promoting antibody production. A schematic representation of the predicted multimeric architecture of the flagellin fusion protein is provided in Figure 1C.

### 3.2 Target gene transfection and protein purification

**Figure 2:**
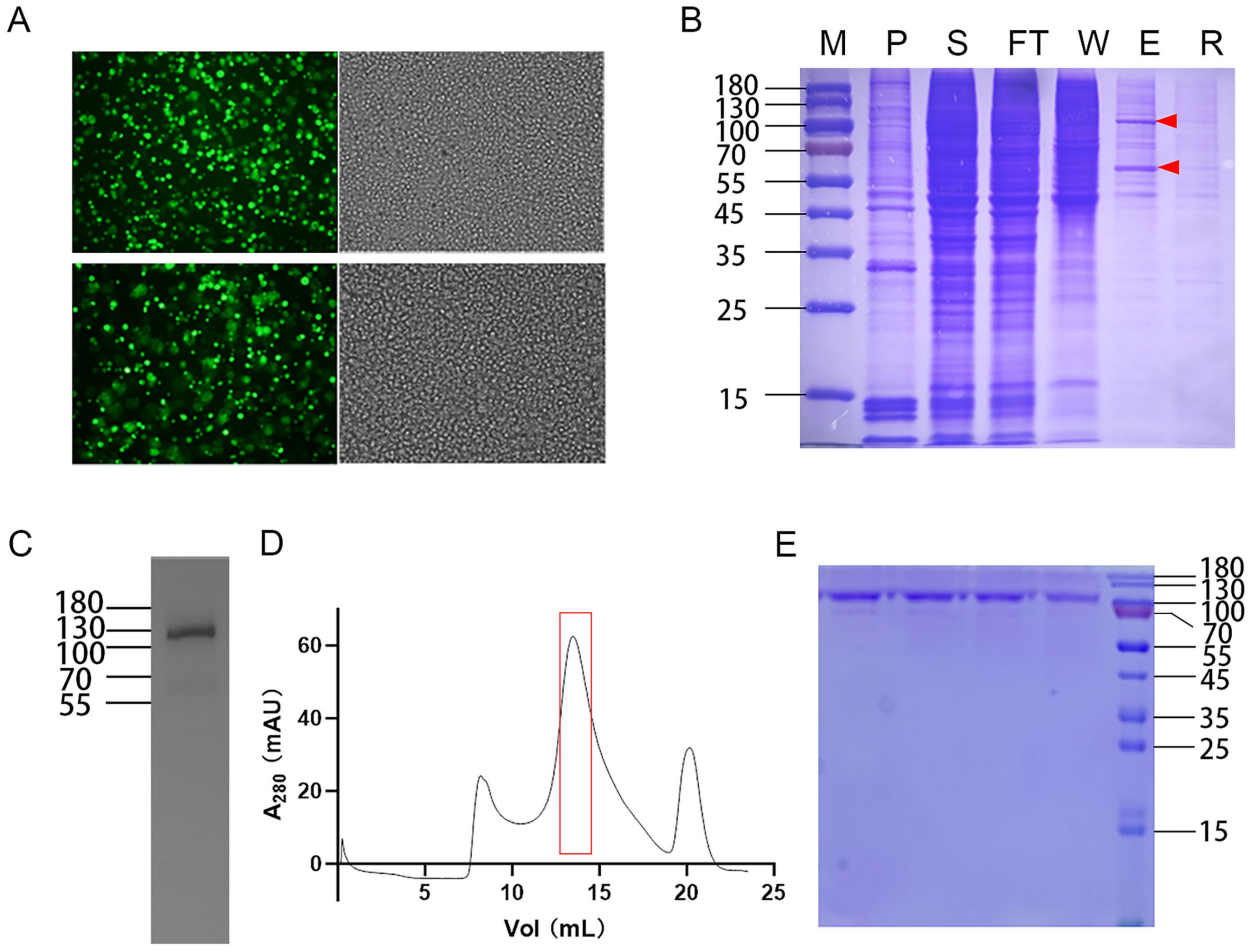
Results of fusion protein transfection and purification **(A)** Fluorescence microscopy images of CHO-S cells at 60 h post-transfection with the target protein construct. Left panel: GFP fluorescence field; right panel: bright-field view. **(B)** SDS-PAGE analysis of the target protein purified by affinity chromatography. M: protein molecular weight marker (kDa), 3 μL loaded; P: pellet after cell disruption and centrifugation, 2 μL loaded; S: supernatant after cell disruption and centrifugation, 2 μL loaded; FT: Ni-NTA column flow-through, 2 μL loaded; W: Ni-NTA column wash fraction, 30 μL loaded; E: Ni-NTA column eluate, 40 μL loaded; R: Ni-NTA resin suspension after elution, 5 μL loaded. **(C)** Western blot identification of the target protein in the eluate fraction. **(D)** SEC elution profile of the target protein purified on a Superose 6 10/300 gel filtration column. The peak corresponding to the target protein is highlighted by the red box. **(E)** SDS-PAGE analysis of the fractions corresponding to the red-boxed peak in panel **(D)**.

The recombinant fusion protein was expressed using the pCAG vector. To facilitate rapid assessment of transfection efficiency, an Enhanced Green Fluorescent Protein (EGFP) tag was fused to the N-terminus of the target protein via a Tobacco Etch Virus (TEV) protease cleavage site. As shown in Figure 2A, fluorescence microscopy based on GFP signal indicated a high transfection efficiency of approximately 80%. Following transfection, the CHO-S cells were harvested between 60 and 72 h for protein purification.

SDS-PAGE analysis revealed the presence of two protein bands in the vicinity of the expected molecular weight (indicated by red triangles in Figure 2B). To further characterize these bands, Western blot analysis was performed, which confirmed that only the upper band corresponded to the target protein, whereas the lower band likely represented a degradation product or non-specifically bound protein (Figure 2C). The target protein was subsequently purified by SEC, and the elution profile is shown in Figure 2D. Fractions corresponding to the peak highlighted by the red box were collected and analyzed by SDS-PAGE (Figure 2E), which verified that this band indeed represented the target protein. Based on its elution volume, the protein likely exists in an polymerized or oligomeric form. The final purified protein was obtained with high homogeneity and is suitable for subsequent functional and immunogenicity studies.

### 3.3 Mouse immune experiment

Given the short experimental cycle, relatively low cost, and well-established reference value of mice as a model organism, this study employed a mouse model to evaluate the immunogenicity of the purified protein. Following protein preparation, the vaccine was formulated using a water-in-oil-in-water (W/O/W) adjuvant 201. The FliC_L3A_-COE vaccine emulsion was prepared by rotor-stirring homogenization and subsequently used for mouse immunization experiments. Mice aged 6–8 weeks were selected for injection. A commercial bivalent inactivated vaccine KXN against PEDV and transmissible gastroenteritis virus (TGEV) (strains AJ1102 + WH-1; Wuhan Keqian Biology Co., Ltd.), which also employs a W/O/W adjuvant and currently holds the largest market share among PEDV preventive vaccines in China, was used as the positive control.

**Figure 3:**
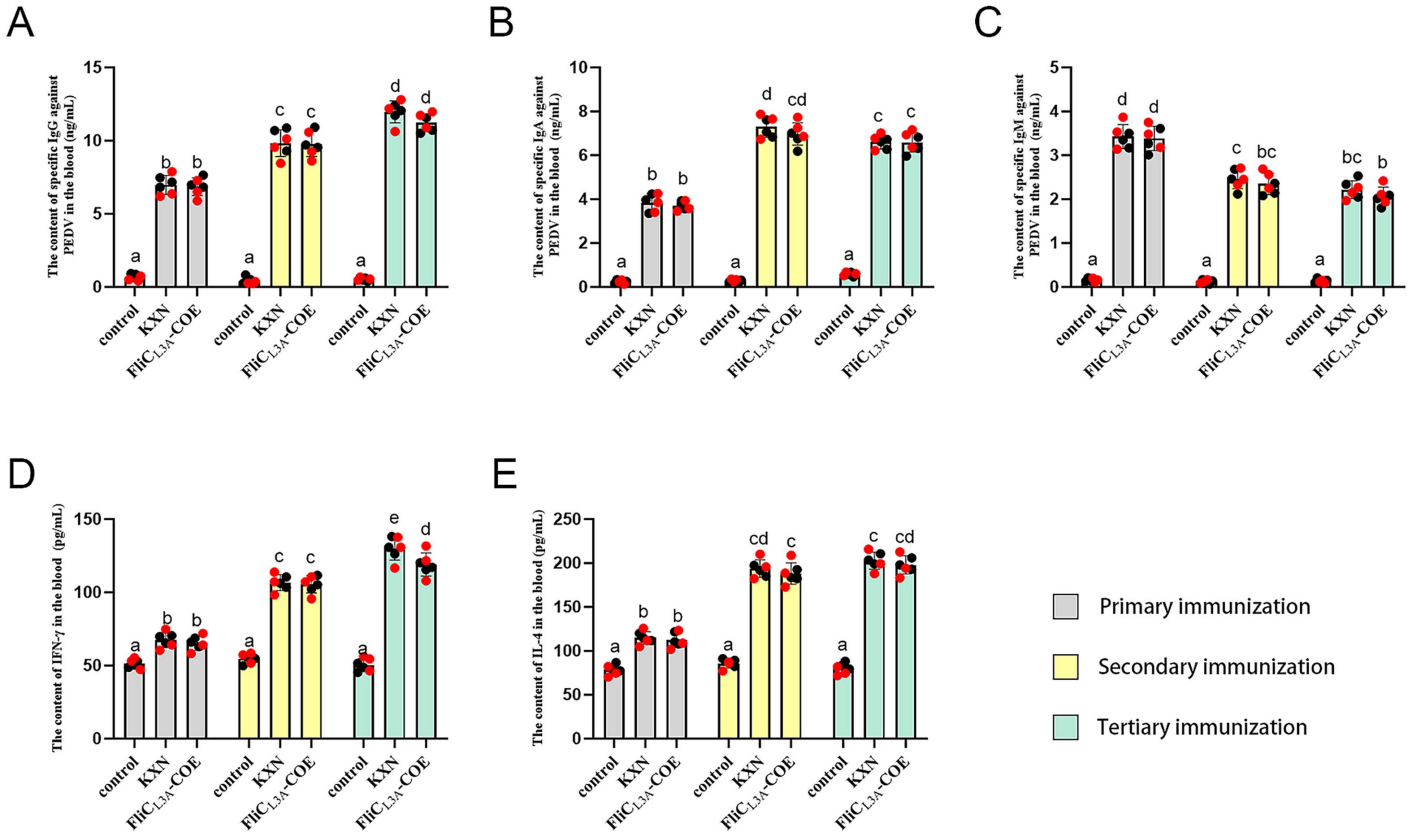
Results of the mouse immune experiments. **(A)** Detection of anti-PEDV IgG antibody levels in mouse serum. **(B)** Detection of anti-PEDV IgA antibody levels in mouse serum. **(C)** Detection of anti-PEDV IgM antibody levels in mouse serum. **(D)** Detection of IFN-*γ* levels in mouse serum. **(E)** Detection of IL-4 levels in mouse serum. Control, negative control group; KXN, commercial vaccine positive control group; FliC_L3A_-COE, experimental group. Groups sharing the same letter are not significantly different, whereas groups with different letters are considered significantly different; *n* = 6, *p* ≤ 0.05. Error bars represent ± SD. Red and black data points denote independent experimental batches, and no significant difference was observed between the two batches of the experimental group, indicating the absence of a batch effect (*p* ≥ 0.05).

Detailed information regarding the immunization schedule and injection volumes is provided in the Methods section. Following the completion of the immunization regimen, antibody titers and cytokine production levels were measured; the corresponding results are presented in Figure 3.

Comparative analysis of immune efficacy demonstrated that, with the exception of IFN-*γ*, all immune parameters induced by the FliC_L3A_-COE fusion protein vaccine were comparable to those elicited by the commercial KXN vaccine. Following three immunizations, the level of IFN-γ induced by the FliC_L3A_-COE vaccine was significantly lower than that of the KXN group, although the absolute difference between the two groups was limited. These results suggest that FliC_L3A_-COE is slightly less effective than KXN in activating natural killer cells and T cells to secrete IFN-γ, indicating relatively weaker immunogenicity in this specific aspect, whereas the overall magnitude of the immune response remained largely comparable to that of the control vaccine. Moreover, the temporal trends in immune parameters following booster immunizations exhibited consistency between the two vaccine groups.

IgA, as a critical maternally derived antibody involved in mucosal protection of piglets, plays a central role in conferring resistance to diarrhea. The data presented in Figure 3 demonstrate that no marked increase in PEDV-specific IgA levels was observed within 1 week following the primary immunization; however, the response rapidly peaked after the second booster immunization and subsequently stabilized without further elevation. These findings indicate that FliC_L3A_-COE effectively enhances IgA production and holds considerable potential for inducing mucosal immunity.

The dynamic changes in IgM and IgG antibody titers closely mirrored their established immunological characteristics: IgM, as the predominant antibody of the primary immune response, was rapidly produced during the early phase of immunization to neutralize invading pathogens and subsequently declined as the immune response progressed; in contrast, IgG induction was relatively delayed but was sustained at elevated levels, conferring long-term protective immunity. The capacity of FliC_L3A_-COE to induce both IgM and IgG production indicates that this fusion protein effectively promotes humoral immune responses. Furthermore, the expression levels of IFN-*γ* and IL-4, key immunoregulatory cytokines, exhibited a gradual upward trend with successive immunizations, reflecting a state of sustained immune activation. The elevated expression of IFN-γ and IL-4 observed in the FliC_L3A_-COE-immunized group suggests that this fusion protein possesses robust immunogenicity and can effectively drive the generation of specific antibodies.

## 4 Discussion

PED has consistently posed a significant challenge to the development of the swine industry. The etiological factors contributing to diarrhea in piglets can be broadly classified into infectious and non-infectious categories. Infectious diarrhea is primarily caused by bacterial, viral, and parasitic infections. While bacterial and parasitic diarrheas can generally be controlled through improved husbandry practices combined with pharmaceutical interventions, often achieving favorable recovery rates, viral diarrhea presents a more formidable challenge. Among viral pathogens, PEDV is a major causative agent responsible for epidemic diarrhea outbreaks.

Currently, the primary vaccines available for PEDV prevention are inactivated vaccines and live attenuated vaccines; however, their protective efficacy remains suboptimal. Apart from vaccination, feedback feeding has been employed as a strategy to confer partial protection to piglets by inducing mucosal immunity in sows. Nevertheless, this technique carries inherent biosafety risks and demands considerable operational expertise, limiting its widespread application in commercial swine herds. Consequently, the development of safer and more effective immunization strategies has remained a central focus of PEDV research.

Murtaza et al. (18). employed a fusion protein strategy similar to that utilized in the present study. Through molecular dynamics simulations, they demonstrated that the TLR5 receptor recognizes the flagellin domain within the FliC-COE fusion protein, thereby activating the mucosal immune system. However, their animal experiments revealed that the antibody levels induced by the wild-type FliC-based fusion protein were significantly lower than those elicited by a commercial inactivated vaccine. To address this limitation, the present study adopted a redesigned construct based on the FliC_L3A_ mutant reported by Li et al. (19) and confirmed that FliC_L3A_ retains the capacity to effectively activate the TLR5 signaling pathway within the context of the fusion protein vaccine, exhibiting markedly superior immune-activating effects compared to wild-type FliC. Nevertheless, the level of IFN-*γ* induced by FliC_L3A_-COE after three immunizations remained lower than that of the commercial KXN vaccine, indicating that its immunogenicity still has certain limitations and that the incremental benefit of repeated boosting is restricted. These findings suggest that, based solely on serum antibody and cytokine measurements, the FliC_L3A_-COE fusion protein does not surpass the commercial vaccine in terms of these immunogenicity indicators; rather, it shows a comparable or partially lower profile. Therefore, further optimization of the subunit vaccine’s protein structure is warranted.

In light of practical requirements in swine production, the findings of this study suggest that immunization of piglets against PEDV necessitates a proactive vaccination strategy. Once large-scale PEDV infection has occurred within a herd, the efficacy of subsequent immunization is markedly limited. Clinical observations indicate that piglets infected with PEDV within the first 7 days of life typically succumb within 1 week; therefore, vaccination should be completed 2–3 weeks prior to the anticipated high-risk period of infection. In the present study, a subunit vaccine model was used to examine serum antibody and cytokine levels at various time points following immunization. The results show that the FliC_L3A_-COE fusion protein can induce PEDV-specific IgG, IgA, and IgM responses in mice, as well as detectable levels of IFN-*γ* and IL-4. The temporal patterns of these immune parameters — with IgG increasing after each boost, IgA peaking after the first boost, and IgM declining after the primary response — are consistent with the expected kinetics of a protein immunogen. The observed antibody and cytokine responses provide information on the immunogenicity of this fusion protein in a mouse model. Future studies, ideally including neutralization assays and, if feasible, challenge experiments in the target species, are needed to further assess the vaccine potential of this fusion protein.

## Data Availability

The raw data supporting the conclusions of this article will be made available by the authors, without undue reservation.
