# Supplementary figures and images for: Construction of subunit vaccine by fusing Salmonella flagellin with neutralizing epitopes of porcine epidemic diarrhea virus
Source: Front Vet Sci. 2026 Jul 15;13:1827170. doi: 10.3389/fvets.2026.1827170 (PMC13418404; doi:10.3389/fvets.2026.1827170)

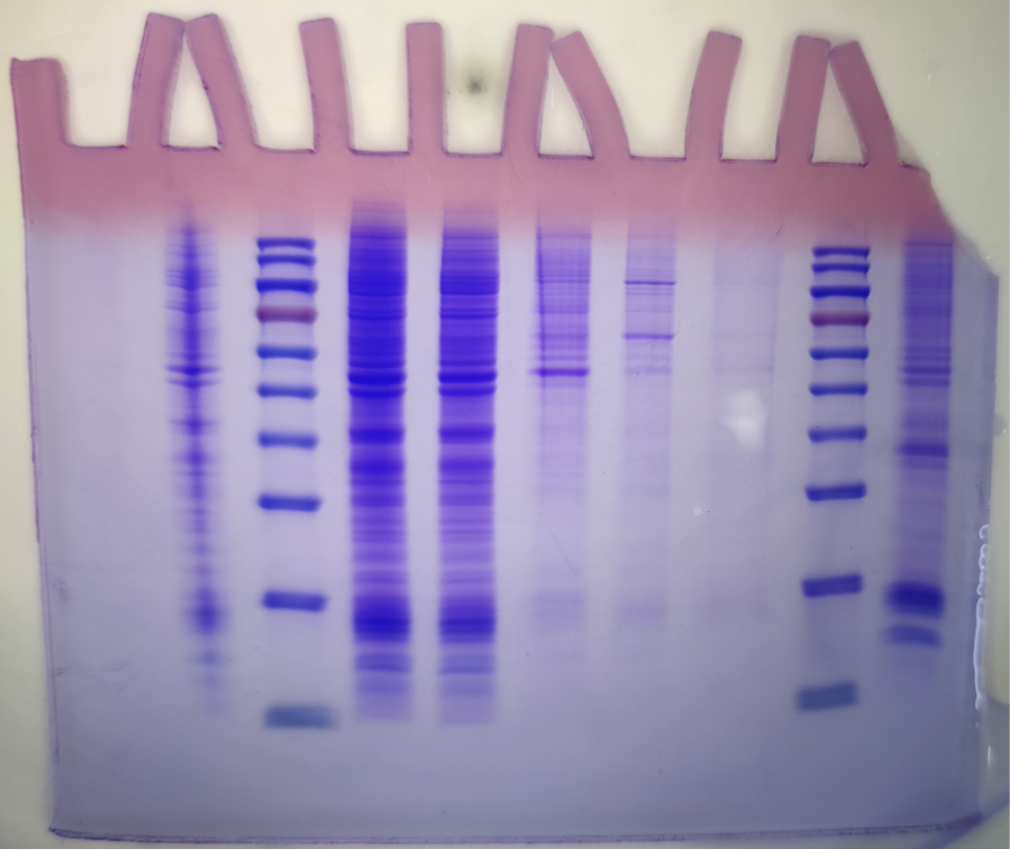

Supplement: Supplementary file 1 [file Data_Sheet_1.ZIP › Original images of SDS-PAGE and Western-blot/Figure 2 B.tif]

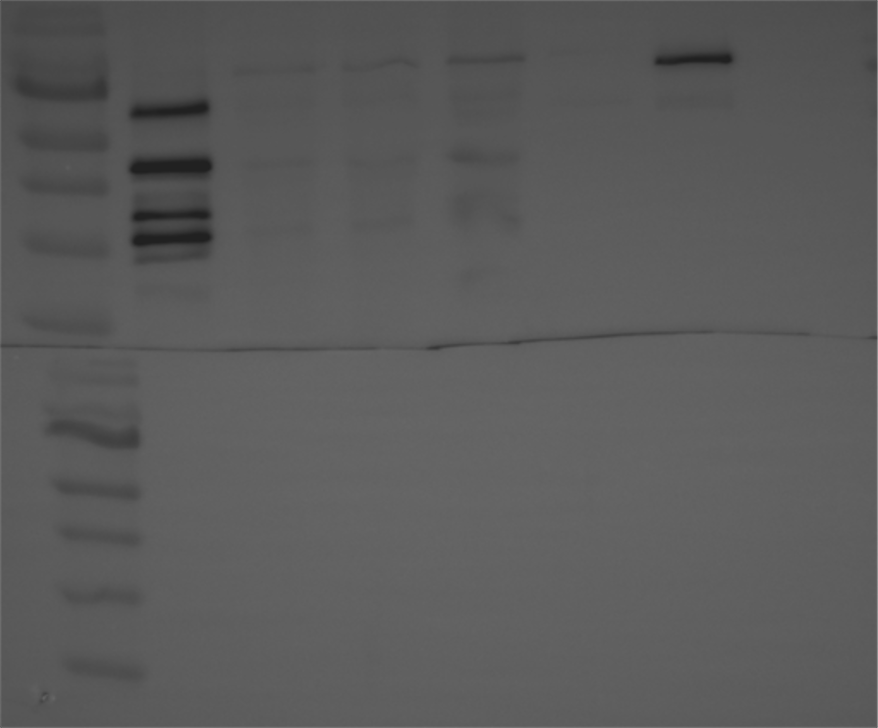

Supplement: Supplementary file 1 [file Data_Sheet_1.ZIP › Original images of SDS-PAGE and Western-blot/Figure 2 C.tif]

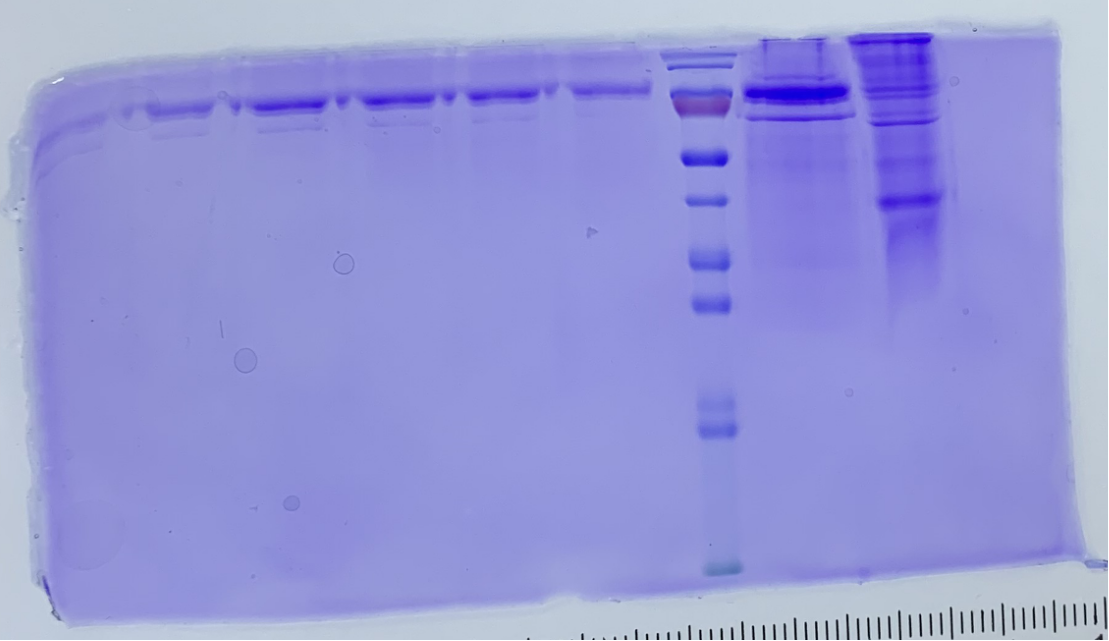

Supplement: Supplementary file 1 [file Data_Sheet_1.ZIP › Original images of SDS-PAGE and Western-blot/Figure 2 E.tif]
